# Oxygen responses within the nucleus accumbens are associated with individual differences in effort exertion in rats

**DOI:** 10.1111/ejn.14150

**Published:** 2018-09-28

**Authors:** Jonathan M. Hailwood, Gary Gilmour, Trevor W. Robbins, Lisa M. Saksida, Timothy J. Bussey, Hugh M. Marston, Francois Gastambide

**Affiliations:** ^1^ Department of Psychology and Behvaioural and Clinical Neuroscience Institute University of Cambridge Cambridge UK; ^2^ Erl Wood Manor Eli Lilly & Co Ltd Windlesham UK; ^3^ Molecular Medicine Research Group Department of Physiology and Pharmacology Schulich School of Medicine & Dentistry Robarts Research Institute Western University London ON Canada; ^4^ The Brain and Mind Institute Western University London ON Canada

**Keywords:** motivation, orbitofrontal cortex, oxygen amperometry, progressive ratio

## Abstract

Goal‐directed motivated behaviour is crucial for everyday life. Such behaviour is often measured, in rodents, under a progressive ratio (PR) schedule of reinforcement. Previous studies have identified a few brain structures critical for supporting PR performance. However, the association between neural activity within these regions and individual differences in effort‐related behaviour is not known. Presently, we used constant potential in vivo oxygen amperometry, a surrogate for functional resonance imaging in rodents, to assess changes in tissue oxygen levels within the nucleus accumbens (NAc) and orbitofrontal cortex (OFC) in male Wistar rats performing a PR task. Within both regions, oxygen responses to rewards increased as the effort exerted to obtain the rewards was larger. Furthermore, higher individual breakpoints were associated with greater magnitude NAc oxygen responses. This association could not be explained by temporal confounds and remained significant when controlling for the different number of completed trials. Animals with higher breakpoints also showed greater magnitude NAc oxygen responses to rewards delivered independently of any behaviour. In contrast, OFC oxygen responses were not associated with individual differences in behavioural performance. The present results suggest that greater NAc oxygen responses following rewards, through a process of incentive motivation, may allow organisms to remain on task for longer and to overcome greater effort costs.

**Abbreviations**
AUC: area under the curve
BOLD: blood oxygen level dependent
CPA: constant potential amperometry
CPE: carbon paste electrode
DA: dopamine
fMRI: functional magnetic resonance imaging
FR: fixed ratio
FSCV: fast scan cyclic voltammetry
NAc: nucleus accumbens
O_2_: oxygen
OFC: orbitofrontal cortex
PIT: Pavlovian‐instrumental transfer
PR: progressive ratio
RDoC: Research Domain Criteria
RPE: reward prediction error

## 1 INTRODUCTION

Motivational impairments are highly prevalent and disruptive in a number of disorders such as schizophrenia (Markou et al., 2013), major depression (Treadway & Zald, 2011), Alzheimer's disease (Lanctôt et al., 2017), Parkinson's disease (Pedersen, Larsen, Alves, & Aarsland, 2009), and Huntington's disease (Naarding, Janzing, Eling, van der Werf, & Kremer, 2009). In spite of the prevalence and severity of these symptoms, currently available treatments have little effect (Fervaha et al., 2015; Seltzer et al., 2004). Recently, the RDoC initiative (Cuthbert & Insel, 2013; Insel et al., 2010) has put forward the idea that transdiagnostic biological domains such as motivation may afford a greater opportunity for therapeutic modulation than existing psychiatric symptom clusters. Thus, an imperative is to gain a more foundational understanding of the neural substrates underlying motivated behaviours in healthy individuals, patient populations, and animal models, using methodology that optimises translation between species.

One important construct involved in expression of motivated behaviours are activational processes that allow organisms to initiate and maintain goal‐directed behaviour and overcome costs or obstacles to achieve goals (Salamone, 1988; Salamone & Correa, 2012). Activational processes appear especially affected in psychopathologies (Salamone, Yohn, López‐Cruz, San Miguel, & Correa, 2016), and as such may represent an important construct that could enable therapeutic opportunities. Such activational processes are often probed in animals and humans through assessment of effort exertion (Markou et al., 2013; Young & Markou, 2015). The prototypical assay of activational processes in rodents is the progressive ratio (PR) schedule of reinforcement (Hodos, 1961). PR schedules probe an organism's ability to maintain responding for reward under increasing effort requirements. Through the use of PR, the neural substrates of effort‐based behaviour have been widely explored (Bailey, Simpson, & Balsam, 2016). Dopaminergic and excitotoxic lesions of the nucleus accumbens (NAc) core can profoundly alter PR performance (Aberman, Ward, & Salamone, 1998; Bowman & Brown, 1998; Hamill, Trevitt, Nowend, Carlson, & Salamone, 1999). The orbitofrontal cortex (OFC) may also be a particularly relevant regional substrate that is not only implicated in a number of neuropsychiatric disorders (Kanahara et al., 2013; Menzies et al., 2008) but also mediates aspects of instrumental behaviour required for PR schedule performance (Cetin, Freudenberg, Füchtemeier, & Koch, 2004; Gourley, Lee, Howell, Pittenger, & Taylor, 2010; Münster & Hauber, 2017).

Concomitantly to measuring direct behavioural changes in effort motivation, neuroimaging, and electrophysiological techniques can be used to assess its underlying neurophysiological correlates. The most valuable approaches in this context are those that afford a cross‐species translation of behavioural and neurophysiological measures. The blood oxygen level dependant (BOLD) contrast measured by functional magnetic resonance imaging (fMRI; Ogawa, Lee, Kay, & Tank, 1990) is a widely used surrogate measure of neural activity in humans. BOLD‐fMRI can be performed in rodents; however, it typically requires rodents to be restrained and/or anaesthetized (Li, Schwarz, & Gilmour, 2016). Constant potential in vivo oxygen (O_2_) amperometry can also be used to assess changes in brain O_2_ levels (Lowry et al., 2010). Crucially, in vivo O_2_ amperometry allows for recording of neurophysiological changes in animals performing complex behavioural tasks (e.g., Li, Martin, Tricklebank, Schwarz, & Gilmour, 2015; McHugh, Fillenz, Lowry, Rawlins, & Bannerman, 2011; McHugh et al., 2014). Furthermore, unlike other preclinical imaging techniques, O_2_ amperometry can provide an adequate (in terms of both validity and viability) proxy measure of the BOLD response in awake rodents (Francois et al., 2016; Howe et al., 2013; Lowry et al., 2010).This allows for the neurophysiological correlates of behaviour to be compared cross‐species.

The aim of the present study was to assess the relative contribution of the NAc and OFC regions to rodent performance under a PR schedule of reinforcement, using O_2_ amperometry. The influence of effort requirements on NAc and OFC O_2_ responses were assessed, as well as the association between individual differences in motivated behaviour and O_2_ responses. Finally, O_2_ responses to rewards delivered independently of behaviour were examined.

## 2 MATERIALS AND METHODS

### 2.1 Animals

Fifteen male Wistar rats (Charles River, UK) took part in this study (mean weight during testing: 610.33 g ± 18.144). All animals were group‐housed (3–4 per cage) throughout the study, in a temperature (20–22°C) and light controlled (lights on 07:00–19:00) environment. All testing took place during the light phase. Animals were given at least 7 days of acclimation in the facility prior to surgical implantation of oxygen recording electrodes. Following a 2‐week surgical recovery period, animals were placed on a schedule of controlled feeding and maintained at no <85% of their free feeding body weight. Water was freely available throughout the study. All experimental procedures were conducted at Eli Lilly and Company Limited in accordance with the Animals (Scientific Procedures) Act 1986 and following approval from the local Eli Lilly Animal Welfare and Ethical Review Board.

### 2.2 Carbon paste electrode construction and in vitro calibration

Carbon paste electrodes (CPEs) were constructed and calibrated in vitro as previously described (Francois, Conway, Lowry, Tricklebank, & Gilmour, 2012; Francois et al., 2014; McHugh et al., 2011) from 8T (200 μm bare diameter; 270 μm coated diameter) Teflon^®^‐coated silver wire (Advent Research Materials, Suffolk, UK). The Teflon insulation was slid along the wire to create a ~2 mm deep cavity, which was filled with carbon paste. Carbon paste was prepared by mixing 7.1 g of carbon graphite powder and 2.5 ml of silicone oil (both Sigma‐Aldrich, O'Neill, Grünewald, Fillenz, & Albery, 1982). All electrodes were soldered to gold connectors. Reference and auxiliary electrodes were also prepared from 8T Teflon^®^‐coated silver wire by removing the Teflon^®^ tip.

In vitro calibration took place within a three‐electrode glass electrochemical cell (C3 cell stand, BASi), with an Ag/AgCl reference electrode and a BASi platinum auxiliary electrode. Calibrations were performed in a 15‐ml phosphate buffered saline solution with a pH of 7.4, saturated with gaseous nitrogen (N_2_), atmospheric air (from a RENA air pump), or pure O_2._ This provided a 3‐point calibration of known concentrations of 0 μM (N_2_ saturated), 240 μM (air saturated), and 1260 μM (O_2_ saturated) oxygen. CPEs were chosen for implantation if their calibration curves were linear and the measured O_2_ values from the saturated solutions were not greatly different from those expected (least square linear regression, *R* ^2^ ≥ 0.98).

### 2.3 Surgical implantation and in vivo validation of carbon paste electrodes

Rats were anaesthetized with 4% isoflurane (1 L/min O_2_) and then maintained on 2% isoflurane (1 L/min O_2_) throughout the surgical procedure. CPEs were implanted into the following regions: bilaterally into the NAc [from bregma: anteroposterior (AP), +1.4 mm, mediolateral (ML), ±1.4 mm and from dura: dorsoventral (DV): −6.1 mm]; and unilaterally into the medial orbitofrontal (mOFC, from bregma : AP, +4.4 mm; ML, +0.6 mm and from dura: DV, −4.0 mm) and the lateral orbitofrontal cortices (lOFC, from bregma: AP, +3.8 mm; ML, +2.6 mm and from dura: DV, −4.4 mm). The reference electrode was implanted, posterior to bregma, into the left posterior cortex (from dura: DV, −1 mm). The auxiliary electrode was secured to a screw positioned, posterior to bregma, above the right posterior cortex. All electrodes were secured with dental cement and the gold connectors inserted into a six‐pin socket (Plastics One), which in turn was cemented into place. Animals received analgesics pre‐ and post‐surgery (carprofen, 5 mg/kg, subcutaneous; Pfizer) as well as antibiotic (Convenia, 5 mg/kg, subcutaneous; Pfizer) administration post‐surgery to aid recovery. Following surgery, animals were placed in thermostatically controlled cages and allowed to regain consciousness.

In vivo validation of the electrodes took place following a 2‐week post‐surgery recovery period by inducing mild hyperoxia and hypoxia through administration of gaseous O_2_ (BOC medical) and nitrogen (BOC gases), respectively. Gases were administered to the animal via a polyurethane tube held ~2 cm from rats’ snouts for 30 s. Three O_2_ and three nitrogen challenges were administered. Validation was considered successful if a positive signal was observed after all O_2_ challenges, but none of the nitrogen challenges_._

### 2.4 Constant potential in vivo oxygen amperometry recording technique

Constant potential amperometry (CPA) was used to measure local event‐related in vivo changes in tissue O_2_, as previously described in detail (Francois et al., 2012, 2014; Lowry, Boutelle, O'Neill, & Fillenz, 1996). Briefly, a constant negative potential (−650 mV) was applied to CPEs to allow for the electrochemical reduction of dissolved O_2_ to occur at the electrode tip. Changes in the measured current are directly proportional to changes in tissue O_2_ (Hitchman, 1978). During each of the recording sessions, rats were tethered to a four channel potentiostat (Biostat, ACM Instruments) via a six‐pin socket and a flexible six‐core cable (both Plastics One). A PowerLab 8/30 Data Acquisition System was used for analogue/digital conversion, and data were collected using Chart v.5 software (AD Instruments) at a sample rate of 200 Hz. Changes in current at each CPE were recorded separately. The negative potential was applied for at least 5 min prior to the start of any behavioural testing. Following each session, raw recordings from each electrode were manually examined, and any sessions displaying gross artefacts, by visual inspection, were removed from future analyses. Event‐related changes in current were analysed according to previous reports (Francois et al., 2012, 2014). Linear interpolation was used to replace occasional missing data points and a biquad Butterworth filter (high pass 0.1 Hz) was used for artefact suppression. Time 0 was taken as the time of reward delivery on completion of each ratio from which time point changes in O_2_ current were measured. To compensate for different baseline between channels, data were normalised to the 1 s period preceding reward delivery. A boxcar‐averaging algorithm was used to down sample the data, keeping a single average from multiple 0.5 s non‐overlapping windows. In the case of the NAc, the signals from the bilateral electrodes were averaged, to create a single NAc response for each animal. As there were no differences in reward O_2_ responses between medial and lateral OFC responses, and to increase the power of the analyses, data from these electrodes were averaged into a single curve for each animal. The area under the curve (AUC) and the peak change in O_2_ response were extracted from each subject's mean O_2_ current.

### 2.5 Behavioural apparatus

Behavioural testing took place within standard rat operant chambers (Med‐Associates, Vermont, USA). Chambers were housed within sound and light attenuating boxes. Each chamber consisted of a house light and two retractable levers either side of food magazine. Standard food pellets (45 mg; BioServ) were delivered to the magazine via an automated dispenser. Experimental sessions were governed by programmes written with Med‐PC software.

### 2.6 Progressive ratio testing

All behavioural testing took place following surgical recovery, 5 days/week. Rats were randomly assigned to either a right or left active lever; this would be the only lever presented throughout the experiment. Training began with one day of magazine training. Over a period of 30 min, food pellets were delivered independently of any behaviour with a variable interval of 60 s (range 15–105 s). Following magazine training, animals began fixed ratio (FR1) training. Each session began with a 30 s of pre‐session blackout, after which the house light was turned on and the lever presented. A single lever press was required for a food pellet to be delivered, and the lever retracted. Following reward delivery, the lever remained retracted for a 15 s intertrial interval (ITI). The session was terminated after either 45 min or following 50 completed trials. All animals were required to complete all 50 rewarded lever‐presses within a session before moving onto the next stage of training. During the next stage of training, five lever presses were required for reward delivery (FR5). As before animals were required to complete 50 trials (250 lever‐presses) before moving onto the final stage of training. All other parameters remained identical to FR1 training. Finally, animals were placed on a PR schedule of reinforcement. Session parameters were identical to the FR1 and FR5 stages, apart from the ITI, which was increased to 30 s. The response requirement on each trial was determined by the following formula: (5 * e^(0.2*n)^−5); where *n* is the trial number, resulting in response requirements of: 1, 2, 4, 6, 9, 12, 15, 20, 25, 32, 40, etc. All animals were initially trained for 5 days without any O_2_ recordings, to ensure a stable performance. Then rats underwent 5 days of habituation to the tethering and recording procedures to ensure no adverse effects of the tethering procedure. During these sessions, PR and CPA recordings were performed as normal, however the data were not analysed. Following this period, animals received 10 sessions of PR (1 session/day), from which O_2_ responses were analysed. The primary behavioural measure of interest was breakpoint, defined as the number of lever presses completed in the last successfully completed trial. Additional measures of behaviour included the rate of lever presses and the delay‐to‐reward (the mean latency from trial start to completion).

### 2.7 Whole session O_2_ amperometry analysis

As slow drifts in baseline over the course of a PR session may confound event‐related analyses, the temporal profile of O_2_ signals was assessed for any absolute changes in global current in either the NAc or OFC. Following the first lever presentation, the change in current was divided into 120 s non‐overlapping bins. Animals are typically only actively engaging in the task for a portion of the 45‐min PR session (i.e., the period prior to breakpoint being reached). Therefore, the absolute change in global O_2_ levels was examined only for the rats’ mean active period, which was calculated by as the time from session onset to the completion of the final trial within a session.

### 2.8 Progressive ratio and event‐related O_2_ amperometry analysis

During the PR task, changes in the recorded current were assessed for a period of 30 s following reward delivery (at the successful completion of every ratio). O_2_ responses were averaged, within regions, to create a single reward response per region per subject. The influence of effort requirements on O_2_ responses was assessed by dividing each session in half based on the number of trials completed. The first half of trials or “early trials” were deemed “low effort” whereas the latter half or “late trials” were deemed “high effort” trials; regardless of the total number of trials completed (e.g., Covey, Dantrassy, Zlebnik, Gildish, & Cheer, 2016; Wanat, Kuhnen, & Phillips, 2010). O_2_ reward responses following low and high effort trials were averaged across the 10 sessions to give a single low and single high effort response per region per subject. Individual differences in motivated behaviour were obtained by dividing the animals into high and low performing rats based upon a median split of the mean breakpoint across the 10 PR sessions. O_2_ reward responses were then compared between these two groups of low and high performing rats. To account for differences in the number of trials completed between these two groups, a separate group comparison was conducted on the minimum number of trials completed by all subjects across all sessions. Behavioural stratification also took place based upon median splits of the mean delay to reward and the mean response rate across the 10 PR sessions, following which O_2_ reward responses were compared between high and low delay and response rate groups.

### 2.9 Non‐contingent reward delivery testing

It is possible that the vigorous, repetitive lever pressing that occurs during PR performance may confound the O_2_ signal analysis. Therefore, following completion of PR testing, animals were exposed to a single session, where food pellets were delivered independently of any behaviour. Small (1 pellet) or large (3 pellets) rewards were delivered pseudo‐randomly without any cue, according to a 120 s variable interval schedule (range 90–150 s).

### 2.10 Non‐contingent reward O_2_ amperometry analysis

For the NAc, O_2_ signals were again examined for 30 s following reward from a 1 s pre‐reward baseline. O_2_ signals from OFC electrodes were analysed for 45 s following reward, as O_2_ levels remained elevated 30 s post‐reward. O_2_ signals in response to small and large rewards were analysed separately. Additionally, O_2_ changes following small and large rewards were examined in the previously identified high and low performing groups. Finally, O_2_ responses to single pellet rewards were compared in the first half and second half of trials_._ This was in order to examine, whether in the absence of any behaviour, there was any temporal‐dependent change in the magnitude of OFC and NAc O_2_ responses.

### 2.11 Histology

In order to confirm CPE placement, rats were euthanized and brains were rapidly removed and placed in 10% buffered paraformaldehyde solution prior to histological analysis (Covance, USA). Brain sections (200 μm) were viewed microscopically to view location of electrode tips; any animal with improper electrode locations was excluded from the analysis.

### 2.12 Statistical analysis

In cases where O_2_ data were excluded (due to excessive noise), the behavioural data were also excluded. Therefore, the stability in breakpoints across the 10 PR sessions was assessed using a mixed model design, which allows for missing cases. A Sidak correction for multiple comparisons was applied during post hoc testing. All O_2_ analyses were completed separately for the NAc and OFC regions. The temporal stability of the O_2_ signal was assessed by averaging each subject's global O_2_ signal across sessions, to give a single global signal per subject. Repeated measures ANOVA was used to test for any significant within‐session change in O_2_ signals. When appropriate, a Greenhouse‐Geisser correction was applied to correct for violations in sphericity. Examining the influences of work‐requirement upon O_2_ responses to reward was achieved by dividing each subject's trials into early (low effort) and late (high effort) trials based upon the number of trials each subject completed in a session. Each subject's O_2_ reward responses were averaged across trial type and session to give a single O_2_ reward responses per subject for both early and late trial types. The time course of these O_2_ reward responses were analysed using a repeated measures ANOVA, with a Fisher’s correction applied to post hoc comparisons. The parameters of each subject's O_2_ response (AUC and peak value) for early and late trials were then compared via within‐subject paired *t* tests.

The effects of individual differences in PR performance and O_2_ responses were tested by arbitrarily splitting animals into high and low performers based upon a median split of the mean breakpoints from across the 10 PR sessions. The time courses were again analysed using a mixed model ANOVA, with a Fisher's post hoc test. The parameters of each subject mean O_2_ reward response from all trials were then compared by high and low responding groups via independent *t* tests. The same between‐subject approach was taken when assessing subjects’ O_2_ responses based upon their mean response rate and mean delay‐to‐reward for each session, where parameters of the O_2_ reward response were compared between groups created by median splits of the mean delay‐to‐reward and response rate. Behavioural measures were compared between high and low responders and between early and late trials and analysed via repeated measures ANOVAs. In the case of the non‐contingent reward delivery testing, O_2_ reward responses to one and three‐pellet rewards were compared within and between high and low performing groups. Repeated measures ANOVAs were used to test for significance, with a Sidak correction applied to any post hoc test. For all statistical tests a significance criterion of *p *<* *0.05 was adopted. All statistics were conducted using SPSS version 23.0 (IBM Corp).

## 3 RESULTS

### 3.1 Histology

**Figure 1:**
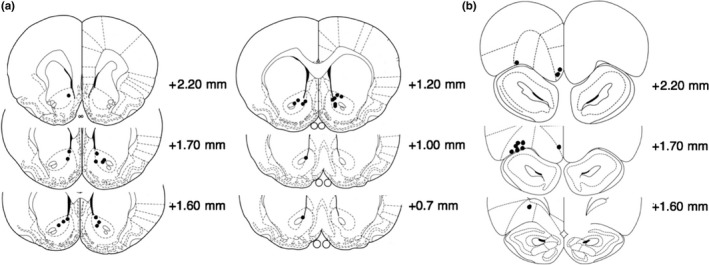
Reconstructions of carbon paste electrodes (CPE) placements within: (a) the nucleus accumbens (NAc) and (b) the orbitofrontal cortex (OFC). The location of CPE tips are marked by the black circles. Coronal slices are adapted from (Paxinos & Watson, 2009)

Figure 1 shows the locations of the NAc (Figure 1a, *n* = 15 rats) and OFC (Figure 1b, *n* = 10 rats) CPEs. For the NAc, bilateral recordings were taken from 10 rats and an additional five rats which only had one single working electrode. As for the OFC, three rats had recordings from medial areas, six from lateral areas, and one with recordings from both the medial and lateral areas.

### 3.2 Behavioural analysis

**Figure 2:**
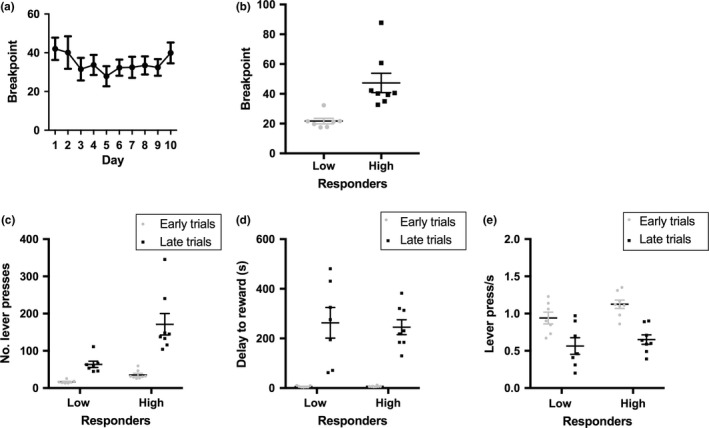
Overview of behavioural performance (a,b) and stratification of behavioural measures by trial type and response groups (c,e). (a) The group level mean breakpoint across the 10 PR sessions. (b) Division of high and low responders based upon a split of subjects’ mean breakpoint. (c) The mean number of lever presses made were greater in late trials, and in high responding rats. (d) The mean delay‐to‐reward was significantly greater in late trials, but did not differ between groups. (e) The mean rate of lever pressing was reduced in late trials, but did not differ between groups. Error bars represent ±1 *SEM*

For analysis purposes, O_2_ responses following reward delivery were collapsed across 10 PR sessions. Therefore, the stability in behavioural performance across these 10 sessions was examined (Figure 2a). There was a significant effect of session upon breakpoint (*F*_1,9_ = 2.248, *p *=* *0.023); however, post‐hoc testing revealed no significant differences in breakpoint between sessions (all comparisons *p *>* *0.05). Additionally, there was only a small degree of variance within‐subjects; the mean range of trials completed (maximum trials completed within a session—minimum number completed) was 3.93 (*SEM* ± 0.45). Together, these analyses suggest that collapsing behaviour (and therefore O_2_ responses) across the 10 PR sessions is a suitable approach.

To assess the relationships between effort and O_2_ reward responses, O_2_ signals were compared between early and late trials as well as low and high responding rats (Figure 2b). Therefore, behavioural differences between these groups were also examined. The mean number of completed lever presses between early and late trials were examined (Figure 2c). There were significant effects of trial type (*F*_1,13_ *= *44.946*, p* *= *0.001) and group (*F*_1,13_ *= *11.873, *p* *= *0.004). There was also a significant trial × group interaction (*F*_1,13_ *= *10.623, *p* *= *0.006). High responding animals made a significantly greater number of lever presses between early and late trials (*p = *0.001), as did low responding rats (*p *=* *0.035). High responding rats also made significantly more lever presses than low performing rats in both early (*p *=* *0.002) and late trials (*p *=* *0.005). It is possible that low and high responding rats also differed on other behavioural parameters. Figure 2d shows the mean delay to reward in early and late trials for low and high responding groups. Delay‐to‐reward increased between early and late trials (*F*_1,13_ *= *56.788, *p* *= *0.001) however, there was no effect of response group (*F*_1,13_ *= *0.065, *p* *= *0.803), nor any group × trial interaction (*F*_1,13_ = 0.078, *p *=* *0.780). The mean rate of responding of high and low responding groups in early and late trials was also examined (Figure 2e). The response rate decreased significantly between early and late trials (*F*_1,13_ *= *217.578, *p *=* *0.001), however, there was no effect of response group (*F*_1,13_ *= *1.610, *p *=* *0.227), nor any group × trial interaction, (*F*_1,13_ = 2.898, *p *=* *0.113).

### 3.3 Within session O_2_ signal stability

Following exclusion of trials and/or sessions containing excessive O_2_ signal artefacts, NAc O_2_ recordings from a total of 143 PR sessions (15 rats, 5–10 sessions per subject) were analysed. The mean active period (latency to last completed ratio) for the NAc rats was 1,041 ± 127.97 s. There was no significant change in current during this period of activity (*F*_1.2,16.9_ *= *2.033, *p *=* *0.171). For the OFC, O_2_ recordings from a total of 83 sessions (10 rats, 5–10 session per subject) were analysed. The mean active period for these rats was 1,207 ± 170.33 s. There was no significant change in current over this period (*F*_1.4,12.7_ = 1.465, *p *=* *0.260). This highlights the stability of the basal O_2_ signal over time and also implies that any event‐related observations were unlikely to be confounded by slow drifts in baseline signal.

### 3.4 Early versus late trial stratification

**Figure 3:**
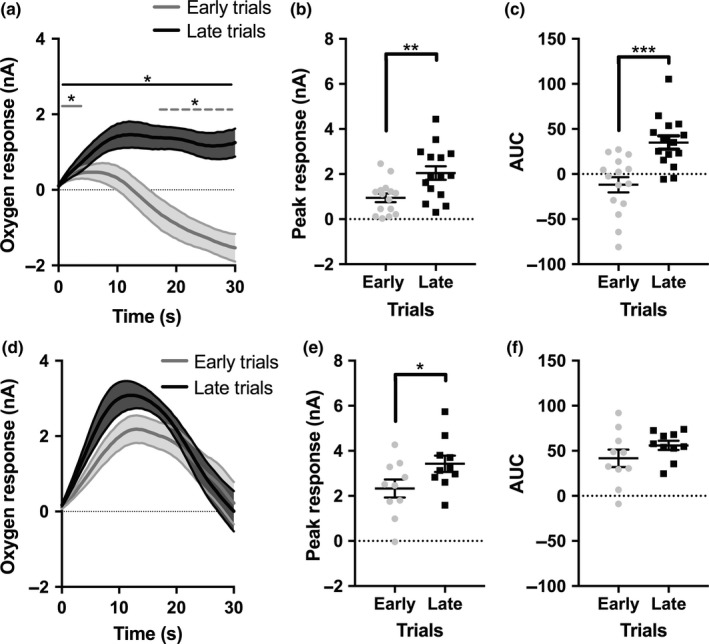
Tissue O_2_ responses within the nucleus accumbens (NAc) and orbitofrontal cortex (OFC) during progressive ratio (PR) performance. (a) Mean O_2_ reward responses within the NAc following the first half (“early”) and second half (“late”) of PR trials across all subjects. The solid grey line represents a significant increase in O_2_ levels in early trials, compared to 0 s. The dashed grey line shows all time points that are significantly lower than 0 s, in early trials. The solid black line shows all time points within late trials with significantly higher O_2_ levels compared to the 0 s time point. (b) The peak NAc O_2_ reward response was significantly greater in late relative to early trials. (c) The area under the curve (AUC) of the NAc response was significantly elevated in late trials relative to the early PR trials. (d) The mean OFC O_2_ reward response following the early and late PR trials across all subjects. (e) The peak value of the mean OFC response was significantly greater in late relative to early trials. (f) The AUC of the mean OFC reward response did not differ between early and late trials. * *p < *0.05; ** *p *< 0.01; *** p < 0.001. Error bars represent ±1*SEM*

Nucleus accumbens O_2_ responses to rewards appeared greater in the late, higher effort trials (Figure 3a). O_2_ responses were significantly affected by both time post‐reward (*F*_1,14_ = 5.463, *p* *= *0.001; partial eta squared *= *0.281) and trial type (*F*_1,59_ *= *34.917, *p* *= *0.001; partial eta squared *= *0.714). There was also a significant interaction between time and trial type (*F*_59,826_ *= *39.408; *p* *= *0.001; partial eta squared *= *0.738). In early trials, there was an initial increase in O_2_ levels. The measured current was significantly greater than baseline for the first 4 s post‐reward (all comparisons *p *<* *0.05, Figure 3a). There was subsequently a significant decrease in O_2_ levels, which were significantly lower than baseline from 18 s to the end of the 30 s trial (all comparisons *p *<* *0.05, Figure 3a). In late trials, there was a significant positive response that was sustained for the duration of the 30 s; all‐time points were significantly greater than baseline (all comparisons *p *<* *0.05). The magnitude of the O_2_ signals was significantly greater in late versus early trials from 6 s post‐reward for the remainder of the 30 s duration (all *p *<* *0.05). For each subject, the peak value and the AUC of the mean NAc O_2_ response were extracted. The peak O_2_ value was significantly greater during late trials (*t*_14_ *= *4.870, *p* *= *0.001; Figure 3b) and AUC (*t*_14_ *= *5.863, *p* *= *0.001; Figure 3c), relative to early trials. This suggests that completion of higher effort trials was associated with greater O_2_ responses to reward. As with the NAc, O_2_ responses to rewards within the OFC appeared greater in later trials (Figure 3d). O_2_ levels were significantly affected by time post‐reward (*F*_59,331_ *= *22.750, *p *<* *0.001; partial eta squared *= *0.717). However, there was no effect of trial type (i.e., early vs. late; *F*_1,9_ *= *2.923, *p* *= *0.121; partial eta squared *= *0.245). There was also no significant interaction between trial type and time post‐reward upon the change in OFC O_2_ levels (*F*_59,531_ *= *1.152, *p* *= *0.213; partial eta squared *= *0.114). The peak OFC O_2_ response was significantly elevated in late trials (*t*_9_ *= *3.636, *p* *= *0.005; Figure 3e); although the difference in the AUC was not significant (*t*_9_ *= *1.760, *p* *= *0.112, Figure 3f).

### 3.5 Low versus high responder stratification

**Figure 4:**
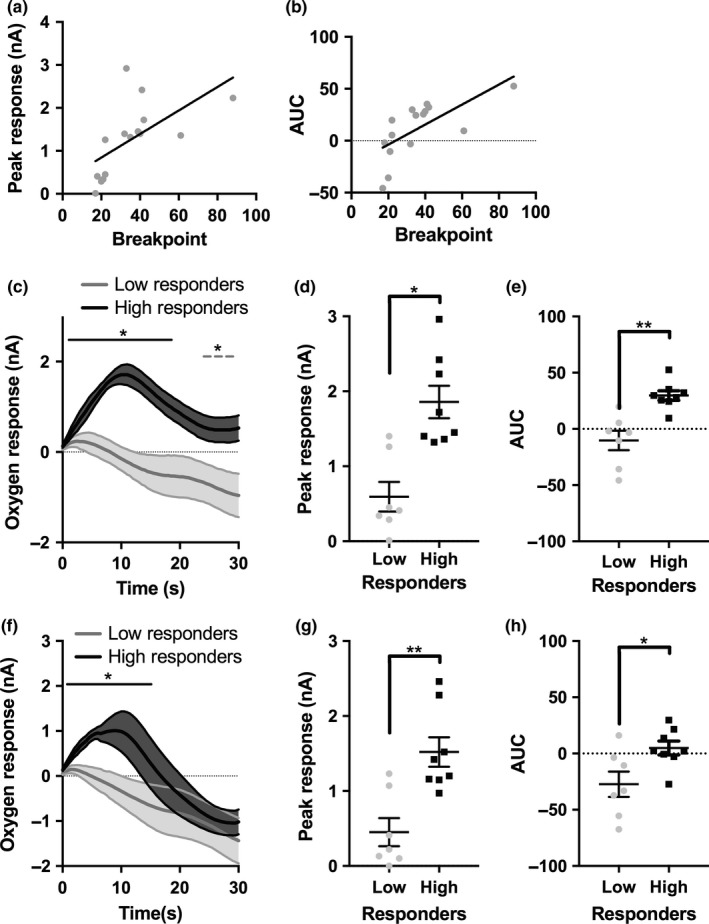
Associations between behavioural performance and nucleus accumbens (NAc) O_2_ responses during progressive ratio (PR) performance. (a) The significant positive correlation between each rats’ breakpoint and peak value of their respective O_2_ response. (b) The significant positive correlation between breakpoint and the area under the curve (AUC) of the NAc O_2_ response. (c) The mean NAc O_2_ reward responses in low and high performing rats. The dashed grey line shows all time points that had significantly lower O_2_ levels relative to 0 s, in low responding rats. The solid black line shows all time points with significantly higher O_2_ levels compared to the 0 s time point, within the high responding group. (d) The peak NAc O_2_ response was significantly greater in high performing rats. (e) The AUC of the O_2_ response was significantly greater in high performers. (f) The mean O_2_ response for high and low performing rats from the first five trials of each session. The solid black line shows all time points with significantly higher O_2_ levels compared to the 0 s time point, within the high responding group only. (g) High performing rats had a significantly higher peak NAc O_2_ response in the first five trials. (h) The AUC of the O_2_ response to reward in high performing rats was also significantly greater in the first five trials. *p < 0.05, **p < 0.01; error bars represent ±1 *SEM*

To examine the relationships between the magnitude of O_2_ responses and behavioural performance, correlations were conducted between the mean breakpoint and NAc O_2_ signal parameters for each rat. Significant positive correlations were observed between mean breakpoint and the peak O_2_ value (*r* *= *0.606, *p* *= *0.017; Figure 4a) and between breakpoint and the AUC (*r* *= *0.672, *p* *= *0.006; Figure 4b) of the NAc O_2_ response, suggesting an association between individual differences in behaviour and NAc O_2_ responses. The relationship between individual differences in behaviour and the NAc O_2_ response was further examined by grouping subjects according to average breakpoint (*n* *= *7 low responders, *n* *= *8 high responders). There was no significant difference in the mean bodyweight of these groups (*t*_14_ *= *1.669, *p* *= *0.119; high responders *= *583 ± 29.89 g; low responders *= *640 ± 12.82 g). The high responding group displayed significantly greater O_2_ responses relative to the low performing group (Figure 4c). The recorded current was significantly affected as a function of time (*F*_59,767_ *= *6.112, *p* *= *0.001; partial eta squared *= *0.320) as well as response group (*F*_1,13_ *= *18.396, *p* *= *0.001; partial eta squared *= *0.586). There was also a significant interaction between time and response group (*F*_59,767_ *= *2.908, *p* *= *0.001; partial eta squared *= *0.183). In low responding rats, there was a significant decrease in O_2_ levels compared to baseline from 24 s post‐reward to the end of the 30 s (*p *<* *0.05). In high responding rats, there was an initial positive O_2_ response, before returning to baseline. The mean time course for high responders displayed a significant positive change in the measured current from 0.5 s post‐reward until 18.5 s post‐reward (all *p *<* *0.05, Figure 4c). Between subjects, high responding rats displayed a significantly greater O_2_ signals, compared to low responders from 3.5 s post‐reward for the remainder of the 30 s analysed (all comparisons *p *<* *0.05). Other O_2_ signal parameters were also significantly different between groups. Both the peak O_2_ response (*t*_13_ *= *4.288, *p* *= *0.001; Figure 4d and the AUC *t*_14_ *= *4.298, *p* *= *0.001; Figure 4e) were significantly greater in high performing rats.

However, this association may be confounded by differences in the number of trials completed between groups. Therefore, an additional between subjects’ analysis of the high and low response groups was limited to the first five trials of each session, the minimum number of trials completed by all animals across all sessions. The high response group still displayed a greater O_2_ response, during these trials, compared to the low response group (Figure 4f). The measured current in the first five trials was significantly affected by both time post‐reward (*F*_59,767_ *= *5.562, *p* *= *0.001; partial eta squared *= *0.300) and response group (*F*_1,13_ *= *6.210, *p* *= *0.023; partial eta squared *= *0.323). There was also a significant interaction between time post‐reward and response group upon the measured current (*F*_59,767_ *= *1.543, *p* *= *0.007; partial eta squared *= *0.106). In low responding rats, no significant positive or negative change from baseline was observed. In high responding rats, there was an early significant increase in O_2_ responses. The measured current was significantly greater than baseline for the first 15 s analysed (all *p < *0.05*,* Figure 4f). No other time points differed significantly from baseline. High responding rats displayed significantly greater O_2_ responses, compared to low responders from 5.5 s to 16 s post‐reward (all *p *<* *0.05). Again, both peak O_2_ response (*t*_13_ *= *3.911, *p* *= *0.002; Figure 4g) and AUC (*t*_13_ *= *2.613, *p* *= *0.021, Figure 4h) were significantly greater in high responding rats.

The time courses for the mean OFC responses following reward were also analysed. The measured current was significantly affected by time post‐reward (*F*_59,472_ *= *21.024, *p* *= *0.001; partial eta squared *= *0.724), but not by response group (*F*_1,8_ *= *0.143, *p* *= *0.715; partial eta squared *= *0.018). There was, however, a significant interaction between response group and time (*F*_29,472_ *= *1.469, *p* *= *0.017; partial eta squared *= *0.281).There was no significant difference between groups at any time point in the measured current (all comparisons *p *>* *0.05). In contrast to the NAc, as shown in Table 1, there were also no significant differences in the extracted measures OFC O_2_ responses between high and low performing groups (AUC: [*t*_8_ *= *0.813, *p* *= *0.440]; peak response: [*t*_8_ *= *0.475, *p* *= *0.647]). There were also no correlations between breakpoint and the parameters of the OFC O_2_ reward response (Table 1).

**Table 1:** The association between behavioural performance and parameters of the orbitofrontal cortex (OFC) O_2_ reward response

OFC	Low responders	High responders	Correlation with breakpoint, *r* (*p‐*value)
Peak response	2.60 ± 0.40	2.92 ± 0.53	0.431 (0.214)
AUC	44.72 ± 10.05	53.00 ± 8.97	0.435 (0.209)

Notes

AUC: area under the curve.

There were no significant differences between low and high responding groups (values are means ± *SEM*) or any significant correlation between breakpoint and either O_2_ reward response parameter.

### 3.6 Delay and response stratification

Alongside increasing response requirements, the delay‐to‐reward concomitantly increases throughout a PR session (e.g., Figure 2b), which may explain the differences in O_2_ responses. To examine the relationship between delay and O_2_ responses, rats were divided into groups, as previously with breakpoint, with longer (*n* *= *8) and shorter delays‐to‐reward (*n* *= *7). NAc O_2_ responses did not significantly differ between these two groups (Peak response: *t*_8.85_ *= *0.205, *p* *= *0.842; AUC: *t*_9.77_ *= *0.305, *p* *= *0.756). Although this cannot exclude the influence of reward delay upon the magnitude of O_2_ reward responses, it suggests the association between individual differences and O_2_ reward responses was not fully mediated by differences in delay‐to rewards. NAc O_2_ signals were also analysed based upon the average rate of responding. As seen in Table 2, there were no significant differences in NAc O_2_ responses between rats with low (*n* *= *7) and high (*n* *= *8) response rates (peak O_2_ response: *t*_7.91_ *= *0.610 *p* *= *0.559; AUC: *t*_13_ *= *1.578, *p* *= *0.139). The association between OFC O_2_ responses and delay‐to‐reward and rates of responding were also examined. Table 2 shows there were no significant group differences, in parameters of the OFC reward response, when rats were grouped by delay‐to‐reward (AUC: [*t*_8_ *= *1.532, *p* *= *0.164]; peak response: [*t*_8_ *= *1.629, *p* *= *0.142]); nor response rate (AUC: [*t*_8_ *= *1.282, *p* *= *0.236]; peak response: [*t*_8_ *= *1.657, *p* *= *0.136]).

**Table 2:** The association between groups based upon median splits of delays‐to‐reward and rate of responding for both the parameters of the nucleus accumbens (NAc) and orbitofrontal cortex (OFC) O_2_ reward responses

	Short delay	Long delay	Slow responders	Fast responders
NAc				
Peak response	1.22 ± 0.15	1.31 ± 0.41	1.12 ± 0.44	1.40 ± 18
AUC	13.40 ± 5.74	9.01 ± 12.47	−0.01 ± 12.35	20.81 ± 6.16
OFC				
Peak response	3.23 ± 0.41	2.21 ± 0.45	2.20 ± 0.44	3.24 ± 0.04
AUC	58.15 ± 9.31	37.42 ± 8.2	38.66 ± 8.80	56.91 ± 9.41

AUC: area under the curve.

There were no significant differences in the parameters of the O_2_ reward response between these groups. Values are means ± *SEM*.

### 3.7 Non‐contingent reward delivery

**Figure 5:**
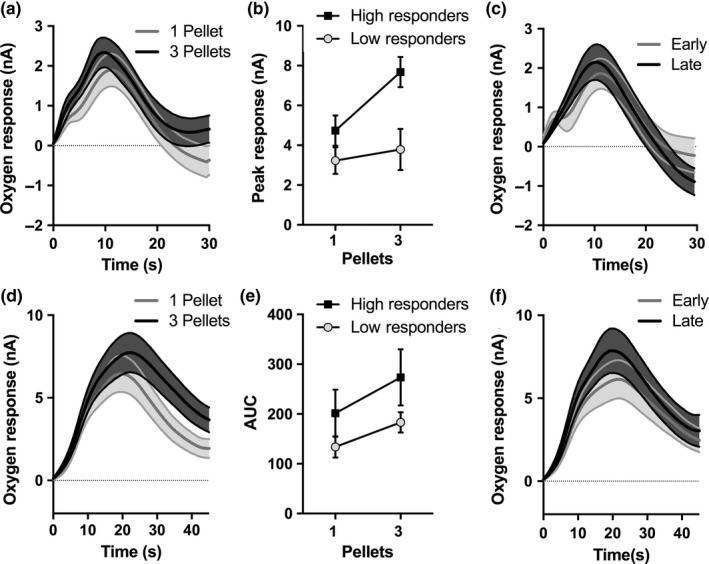
Tissue O_2_ changes following uncued rewards within the nucleus accumbens (NAc) and orbitofrontal cortex (OFC). (a) The mean O_2_ responses to 1 (“small”) and 3 pellet (“large”) rewards within the NAC. (b) The peak NAc O_2_ response of the responses to single and three pellet rewards for both low and high responding animals. (c) NAc O_2_ responses to early and late single pellet reward trials. (d) The mean OFC O_2_ responses to 1 and 3 pellet rewards. (e) The area under the curve (AUC) of the OFC O_2_ response to small and large rewards for both low and high responding rats. (f) OFC O_2_ responses to early and late single pellet reward trials. Error bars represent ±1 *SEM*

To control for non‐specific effects of arousal or that may have existed between response groups, O_2_ responses following rewards delivered independently of behaviour were examined. Delivery of small (1 pellet) and large (3 pellets) rewards elicited strong positive NAc O_2_ responses (Figure 5a). O_2_ responses to small and large rewards were examined based on the previously identified low and high PR responding groups. There were significant main effects of both reward magnitude (*F*_1,13_ *= *7.112, *p* *= *0.029) and group (*F*_1,13_ *= *5.344, *p* *= *0.036) for the peak O_2_ response, but no significant interaction (*F*_1,13_ *= *2.116, *p* *= *0.138, Figure 5b). There were, however, no significant effects of reward magnitude (*F*_1,13_ *= *3.463, *p* *= *0.086); group (*F*_1,13_ *= *161, *p* *= *0.165), nor any interaction (*F*_1,13_ *= *2.258, *p* *= *0.157), for the AUC. Trials were then divided into first half and later half of the trials. NAc O_2_ responses to single pellet rewards did not appear to change between early and late trials (Figure 5c). Neither the peak NAc response (*t*_14_ *= *0.570, *p* *= *0.578) nor the AUC (*t*_14_ *= *1.605, *p* *= *0.131) significantly differed between early and late trials.

Orbitofrontal cortex O_2_ responses to small and large reward delivery were also analysed, as described above. Delivery of small and large rewards resulted in a O_2_ response, lasting longer than previously analysed 30 s period post‐reward delivery. O_2_ responses following delivery of non‐contingent food rewards was therefore analysed over a longer period of time (45 s) (Figure 5d). The peak OFC response was not significantly affected by reward magnitude (*F*_1,8_ *= *2.492, *p* *= *0.153), nor by response group (*F*_1,8_ = 0.168, *p* *= *0.692). There was no significant magnitude × group interaction effect on the peak OFC response (*F*_1,8_ *= *1.915, *p* *= *0.204). There was, however, a significant main effect of reward magnitude (*F*_1,8_ *= *15.91, *p* *= *0.004), on the AUC of the OFC O_2_ response (Figure 5e). However, the AUC was not affected by response group (*F*_1,8_ *= *0.18, *p* *= *0.682), nor any significant magnitude × group interaction (*F*_1,8_ *= *0.006, *p* *= *942). OFC O_2_ responses to small rewards in early and late trials were also examined (Figure 5f). As with the NAc, there were no significant differences between trial types in the magnitude of the OFC O_2_ responses (peak response: [*t*_9_ *= *1.281, *p* *= *0.232]; AUC: [*t*_9_ *= *0.412, *p* *= *0.690]).

## 4 DISCUSSION

Using a highly translatable proxy measure of neural activity, as well as a translational assay of motivation, O_2_ responses to reward within the NAc were predictive of individual differences in motivated behaviour. To our knowledge, no previous study has investigated the association between a functional imaging measure such as this and individual differences in effort‐based behaviour. Furthermore, the assessment of changes in tissue O_2_ allows for direct comparison of results with human BOLD‐fMRI. Functional neuroimaging can be used to help establish equivalence of cognitive processes across species. Whereas there are several rodent imaging techniques that can directly measure electrophysiological and neurochemical correlates of behaviour, it is not typically possible to perform such techniques in humans. Given the widespread use of fMRI in humans, techniques such as O_2_ amperometry, may better help bridge the translational divide, and facilitate basic research into clinical benefit.

In the present study, O_2_ responses to rewards within both the NAc and OFC increased in magnitude as the effort exerted to obtain the rewards grew. These increases in O_2_ responses were not observed in the absence of any work requirements, suggesting that neither accumulation of rewards nor the progression of time was sufficient for an increase in O_2_ responses. Moreover, the magnitude of the O_2_ responses within the NAc was associated with behavioural performance. Rats exerting greater amounts of effort displayed greater NAc O_2_ responses to rewards. This association remained when controlling for the total number of trials completed, highlighting how subjects that show greater NAc activity in early, low effort trials, subsequently continue to overcome greater effort costs, and achieve higher breakpoints. Furthermore, these high performing rats also showed greater NAc O_2_ responses to rewards delivered independently of any behaviour. This observation suggests that the previous results were not a confound resulting from differences in activity levels between groups. In contrast, O_2_ responses within the OFC did not predict PR performance. Consequently, the pattern of activity observed within the NAc displayed at least some degree of regional specificity. Taken together, this suggests that PR performance is directly related to the neural responses to reward within the NAc.

### 4.1 Physiological basis of the measured O_2_ signals

The physiological origin of the O_2_ signal measured within the NAc has been previously discussed in detail (Francois et al., 2012, 2014; Lowry et al., 2010). The measured signal reflects changes in extracellular tissue concentrations of O_2_ (Lowry, Boutelle, & Fillenz, 1997). Increases in hemodynamic measures such as tissue O_2_ concentrations or the BOLD contrast, occur in response to neuronal activation and/or changes in cerebral blood flow. This allows for the use of techniques such as CPA and BOLD‐fMRI as proxy measures of neural activity. Tissue O_2_ concentrations are highly correlated with induced changes in regional cerebral blood flow (Lowry et al., 1997), as is the BOLD signal measured with fMRI (Logothetis & Wandell, 2004). Changes in cerebral blood flow appear primarily related to local synaptic activity (Mathiesen, Caesar, & Lauritzen, 2000). Similarly, BOLD‐fMRI is believed to reflect afferent inputs to an area rather than spiking outputs (Logothetis, Pauls, Augath, Trinath, & Oeltermann, 2001). Together, this raises the possibility that the present changes in O_2_ levels, within the NAc, are driven by afferent inputs to this region. A major input to the NAc, are dopaminergic neurons projecting from the ventral tegmental area. This input pathway has been widely linked with, among other processes, effort exertion. Lesions to this pathway severely disrupt PR performance (Aberman et al., 1998; Hamill et al., 1999; Sokolowski & Salamone, 1998). Furthermore, manipulation of this pathway either via optogenetic or pharmacogenetic tools can bidirectionally affect breakpoints (Boekhoudt et al., 2018; Fischbach‐Weiss, Reese, & Janak, 2018). As with the current O_2_ results, the magnitude of phasic DA reward responses, measured with fast scan cyclic voltammetry (FSCV) are greater in later, higher effort PR trials (Covey et al., 2016; Wanat et al., 2010) whereas, DA responses to rewards following low effort trials are negligible (Wanat et al., 2010). Together, this raises the possibility that the current O_2_ results may reflect changes in DAergic neuron activity within the NAc, however, more appropriate methods (e.g., FSCV) should be used to confirm this.

In the present study, negative O_2_ responses were observed on several occasions, as have been observed in previous studies (Francois et al., 2014; McHugh et al., 2014). O_2_ changes in response to rewards were calculated as a relative change, compared to a 1 s pre‐reward baseline period. During the pre‐reward period, it is likely that the animals would have been actively engaged in lever pressing. A degree of NAc activity, and therefore extracellular O_2_, would be expected during this baseline period. The negative changes observed in the present study, may therefore represent a return to normal levels. In support of this, in the absence of any pre‐reward effort, during the non‐contingent reward paradigm, no negative O_2_ responses were observed.

### 4.2 O_2_ reward responses and individual differences in behaviour

It is noteworthy that high responders still show significant positive NAc O_2_ changes in response to rewards in spite of the likely pre‐reward neural activity. A greater neural response to reward may motivate future behaviour, enabling subjects to remain on task for longer and overcome greater effort requirements. Appetitive rewards, such as food, produce activational effects on behaviour, that can increase the vigour and frequency of behaviour (Skjoldager, Pierre, & Mittleman, 1993) Likewise, enhanced NAc DA release has an activational effect on behaviour (Robbins & Everitt, 1992, 2007). The increased NAc O_2_ responses in high performing rats may, therefore, reflect a greater level of behavioural activation in response to food rewards.

In operant testing, effort is typically modulated by increasing the number of lever responses needed for reward. As a consequence, the delay from trial onset to reward delivery also increases. Reward‐based DA responses have been shown to increase in response to escalating delays (Wanat et al., 2010). DA responses within the NAc have also been shown to signal reward prediction errors (RPE; Schultz, Dayan, & Montague, 1997). The increasing response requirements during PR may result in rewards becoming more unpredictable as a session progresses. The present findings could also be a reflection of greater RPEs in high effort trials. It is not clear, however, how either of these could account for the differences in NAc O_2_ signals between low and high performing animals, since there was no difference in the mean delay‐to‐reward between these two groups. Although we sought to further examine the effects of longer delays as well as changes in RPEs, these should be fully investigated in future and separate studies. A control group with rewards yoked to delivery of rewards on a PR task (e.g., Wanat et al., 2010) would allow us to investigate the role of increasing delays on O_2_ reward responses in the absence of any effort component. The contribution of NAc during expectancy of upcoming rewards has already been investigated using O_2_ amperometry (Francois et al., 2012, 2014). Interestingly, alterations in reward anticipation have previously been linked to motivational deficits in some clinical populations (Barch, Pagliaccio, & Luking, 2016; Wolf et al., 2014), and therefore assessing changes in O_2_ signals pre‐reward delivery could also be of interest as a potential correlate of motivated behaviour.

Alongside regulating effort exertion, a number of studies have also investigated the role of NAc DA in Pavlovian‐instrumental transfer (PIT). During PIT, cues associated with rewards are able to exert strong influences on behaviour, enhancing instrumental responding in their presence. Dopaminergic receptor blockade within the NAc disrupts PIT (Dickinson, Smith, & Mirenowicz, 2000), whereas intra‐accumbens infusions of d‐amphetamine enhances the Pavlovian influences on instrumental responding (Wyvell & Berridge, 2000). FSCV has been used to extend these findings, showing that a reward‐paired cue that generated PIT was accompanied by a phasic DA response (Wassum, Ostlund, Loewinger, & Maidment, 2013). Moreover, the magnitude of the PIT effect was correlated with the magnitude of the phasic DA response (Wassum et al., 2013). When examining the effects of non‐contingent reward delivery in NAc O_2_ responses, it appears that only the high responding rats show an increased magnitude O_2_ response following the three‐pellet reward delivery (Figure 5b). It is worth noting that high responders appeared to only show a greater magnitude reward response following three‐pellet rewards. Since, this probe was conducted after PR training, animals had only been exposed to single pellet rewards. The enhanced increase to these unexpectedly large rewards may be a larger positive prediction error, which may reflect individual differences within the mesolimbic DA system. Furthermore, during this probe, rewards were delivered independently of any instrumental contingency, suggesting the O2 responses reflect Pavlovian influences upon reward. The present association between NAc O_2_ responses and individual differences in PR performance may, therefore, be a reflection of individual differences in Pavlovian influences on behaviour or incentive motivation. In other words, the greater incentive motivation enabled the high performing rats to overcome greater effort costs to obtain more rewards under the PR schedule of reinforcement.

### 4.3 The role of the OFC in effort‐related behaviour

The use of a control region is important in the study of O_2_ responses, to demonstrate that any results are not caused by some global change in tissue O_2_ levels that may confound the results. This may be especially important when using a behavioural assay such as PR, which involves a large amount of vigorous, repetitive responding. In the present study, we used the OFC as a control region and we concluded that the effects observed within the NAc were regionally specific, as there was no association between behavioural performance and OFC O_2_ reward responses. We also observed no difference in the O_2_ reward responses between electrodes placed in the medial and lateral regions of the OFC. However, previous reports have demonstrated that these regions of the OFC are functionally distinct (Noonan, Kolling, Walton, & Rushworth, 2012, for a review). Within the lateral OFC, excitotoxic lesions do not affect breakpoints in either rats (Kheramin et al., 2005) or mice (Gourley et al., 2010). In contrast, excitotoxic lesions to the medial OFC result in an increase in breakpoints in rats (Münster & Hauber, 2017) and mice (Gourley, Kiraly, Howell, Olausson, & Taylor, 2008), resembling the effects of excitotoxic lesions to the NAc (Bowman & Brown, 1998). The medial OFC has strong projections to the NAc core (Hoover & Vertes, 2011). In contrast, the lateral OFC projects mainly to dorsolateral regions of the striatum (Schilman, Uylings, Galis‐de Graaf, Joel, & Groenewegen, 2008), an area that is not involved in supporting PR performance (Eagle, Humby, Dunnett, & Robbins, 1999). Together, these studies suggest the medial, but not the lateral, OFC regulates effortful instrumental responding. Presently, the majority of the CPEs were located within the lateral OFC (Figure 1b), which may explain the lack of association between OFC O_2_ recordings and behavioural performance.

Orbitofrontal cortex O_2_ responses did, however, increase between early and late PR trials. It is unlikely that this effect represents RPE signals as OFC activity is not well correlated with RPEs (Hare, O'Doherty, Camerer, Schultz, & Rangel, 2008). The lateral OFC has been widely implicated in modulating delay‐based responding (Winstanley, Theobald, Dalley, Cardinal, & Robbins, 2006; Zeeb, Floresco, & Winstanley, 2010). Lesions to the lateral OFC reduce an animal's ability to tolerate delays for larger rewards (Mar, Walker, Theobald, Eagle, & Robbins, 2011) and levels of the DA metabolite DOPAC, increase within the lateral OFC, during a delay‐discounting task (Winstanley et al., 2006). The increased O_2_ reward responses in late trials may, therefore, have been a reflection of the greater delay‐to‐reward experienced in those trials. OFC O_2_ signals were also modulated by reward outcome. Lateral regions of the OFC encode information regarding reward magnitude during reward receipt (van Duuren, Lankelma, & Pennartz, 2008; van Duuren et al., 2007) suggesting, CPA can accurately capture OFC activity during processing of reward.

## 5 CONCLUSIONS

Amperometric measurement of tissue O_2_ changes, a highly translatable and valid proxy measure of BOLD‐fMRI in behaving rodents, provided a novel insight into the role of the NAc function and individual differences in effort‐related behaviour. The O_2_ response to reward within the NAc is related to effort exerted under a PR schedule of reinforcement. This highlights the dynamic role neural signals within the NAc play in maintaining motivated behaviour. Furthermore, this study demonstrates in vivo O_2_ amperometry can be used to probe the neural correlates of behaviour in rodents. Furthermore, through the use of such techniques, hypotheses can be derived that can subsequently be tested in humans, therefore facilitating cross‐species research.

Within the RDoC framework (Cuthbert & Insel, 2013) aberrant approach motivation has been identified as a transdiagnostic symptom of psychiatric disorders. One subconstruct of motivation within this framework is effort valuation/willingness to work. The use of PR schedules can be used, across species, to probe these subconstructs of motivated behaviour (Young & Markou, 2015). The approach taken by the RDoC initiative emphasises the need to bypass diagnostic categories and first understand the neural substrates of behavioural constructs in both healthy and non‐healthy subjects. Therefore, the present study is in line with this approach. Through identifying a translatable imaging correlate of PR performance, future studies could use fMRI to determine whether there is an equivalent association between the BOLD response and PR performance in humans and whether this association is disrupted in clinical populations.

## CONFLICT OF INTEREST

GG and HMM are employees of Eli Lilly and Co. FG was an employee of Eli Lilly and Co. at the time of research. TWR discloses consultancy with Cambridge Cognition, H. Lundbeck A/S, Unilever, and Mundipharma and has research grants with H. Lundbeck A/S and Shionogi. LMS and TJB consult for Campden Instruments, Ltd.

## DATA ACCESSIBILITY

Data are available upon request from the corresponding author.

## AUTHOR CONTRIBUTIONS

JMH and FG: Designed the experiment, performed the research; analysed the data and wrote the manuscript. GG: Analysed the data and wrote the manuscript. TWR, TJB, and HMM: wrote the manuscript.
