# Supplementary material for: Oxygen responses within the nucleus accumbens are associated with individual differences in effort exertion in rats
Source: Eur J Neurosci. 2018 Sep 28;48(9):2971–87. doi: 10.1111/ejn.14150 (PMC6282581; doi:10.1111/ejn.14150)
Supplement: Supplementary file 1 [file EJN-48-2971-s001.docx]

Oxygen responses within the nucleus accumbens are associated with individual differences in effort exertion in rats

Hailwood, Jonathan; Gilmour, Gary; Robbins, Trevor; Saksida, Lisa; Bussey, Tim; Marston, Hugh; Gastambide, Francois

_____________________________________________________________________________________

| Review timeline: | Submission date: 04/05/18  Editorial Decision: Minor Revision  Revision Received: 14/08/18  Accepted: 10/09/18 |  |
| --- | --- | --- |
|  |  |  |

Editor: Anthony Isles

Reviewer 1: Daniele Caprioli

Reviewer 2: Josephine Haddon

_____________________________________________________________________________________

1st Editorial Decision	31/07/18

_____________________________________________________________________________________

Oxygen responses within the nucleus accumbens are associated with individual differences in effort exertion in rats

Dear Dr. Hailwood,

First of all, apologies for the time it has taken to deal with you manuscript which was due to difficulties in engaging reviewers.   It has now been seen by two external reviewers as well as by the Section Editor,  Dr. Anthony Isles, and ourselves and the good news is that they like the study and suggest only minor revisions.

As you can see below, although reviewer 1 suggests shortening the discussion, reviewer 2 raise a longer series of points than can mostly be addressed by clarification of the text and possibly some additional analysis.  Please carefully address each point that he/she has raised in a revised version of the manuscript.  In accordance with EJN guidelines, please also replace bar chart with more informative scatter plots, hybrid plots or similar.

When revising the manuscript, please embolden or underline major changes to the text so they are easily identifiable and please don't leave 'track change' formatting marks in your paper.  Please ensure that you provide a text and a figure file for the Graphical Abstract (as detailed in the instructions below).  When carrying out your revisions please refer to the checklist below and visit the EJN author guidelines at www.ejneuroscience.org

When finalized, please upload your complete revised manuscript onto the website, as a Word file (.doc, or .docx).  Please also ensure that a complete set of tables and figures is included as separate files, even if these have not changed from the originals.  At this stage it is necessary to provide high resolution figures.  Please see important instructions below.

Please go into https://mc.manuscriptcentral.com/ejn - Author Centre - manuscripts with decisions where you will find a 'create a revision' link under 'actions'. We ask that you please indicate the way in which you have responded to the points raised by the Editors and Reviewers in a letter. Please upload this response letter as a separate Word (.doc or PDF) file using the file designation "Authors' Response to Reviewers" when uploading your manuscript files.  Please DO NOT submit your revised manuscript as a new one.  Also, please note that only the  Author who submitted the original version of the manuscript should submit a revised version.

If you are able to respond fully to the points raised, we would be pleased to receive a revision of your paper within 30 days.

Thank you for submitting your work to EJN.

Best wishes,

Paul Bolam & John Foxe
co-Editors in Chief, EJN


Reviews:

Reviewer: 1

Comments to the Author
This is an interesting paper in which Hailwood and colleagues, showed that oxygen responses within the nucleus accumbens, but not the orbitofrontal cortex, were associated with individual differences in effort exertion in rats. To this aim the authors used constant potential in vivo amperometry in rats performing a progressive ratio task. Furthermore, the authors put in place adequate control to exclude possible confounds.  

The experiments are well conceived and clearly described. Overall, the manuscript is well written and easy to follow. The authors provided an interesting and thorough discussion.

I have just a suggestion.

By shortening the discussion (thorough but lengthy), the authors should save some space where to discuss explicitly the study into the RDoC perspective (dedicated section). I believe that this already excellent study would benefit from it.


Reviewer: 2

Comments to the Author

The authors report an interesting study examining oxygen responses in the nucleus accumbens(NAc)  and orbital frontal cortex (OFC) during progressive ratio (PR) task in rats, demonstrating increased oxygen response in the NAc which they conclude is a consequence of increased effort on the task. I think the topic is of interest to the audience of EJN but the results section in particular can get a bit confusing. The authors report several analyses with many different behavioural and O2 measures, and it is not always clear what data the analyses are conducted on. This, combined with errors and omissions in the data makes the findings hard to assess appropriately.  I believe, however, that with further revision the manuscript would be suitable for publication.


1. Cannulae were implanted into 2 regions of the OFC unilaterally. Why were these not bilateral implantations. Moreover the different cannulae implantations  into medialorbital and lateral regions were not used – instead recordings from both were combined (although there are fewer in the lateral OFC). Why was this the case? And are there any differences in recording from the regions of the OFC? This needs to be addressed in the manuscript.

2. Stability of behavioural performance ( p13 and Figure 2)The authors report that high responders perform more lever presses during both early and late trials than low responders, but there were no reported differences between high/low responders on delay to reward, or on lever press/sec  – I’m not sure how this can be the case if the high responders are responding more? The high responders are at a higher breakpoint (FR) in which case there should be a longer delay to reward if responding at the same rate as the low responders. Unless (p10 line 233) median splits are performed separately for each measure – although I’m not sure of the logic for this.
It is also important to also report the breakpoints of these groups rather than just the mean lever presses.

3. Within session O2 signal stability –p14 line 319 onwards. The authors report NAc O2 recordings from a total of 143 PR sessions (from 15 rats), wheres OFC reacordings were from 83 sessions (and only 10 rats). Why were there differences in sessions/rats from each of the regions? And did all 10 animals contribute to recordings from both regions of the OFC?
In addition – the behavioural data reported was for all 10 sessions, for all 15 rats – was there any variability in behavioural performance when you use only the sessions that were included in the O2 analysis?

4. Early vs Late Trial stratification p14 line 330 onwards. Has the sub-baseline O2 response been observed in other studies? Or is the baseline not meaningful in early trials due to greater variability in behavioural response?

5. Low vs High response stratification p15. I think you are missing some figures, or there is an error – Para starting p17 line 393 references figure 4G and 4H as showing the data for the OFC, however these figures have already been referred to in line 391 as showing data from the NAc for the first 5 trials of the session, thus these data are hard to interpret.


6. The effects of non-contingent reward delivery on O2 levels was examined – specifically comparing the effect of 1 vs 3 pellet delivery – to account for non-specific effects of arousal .
a. The authors report that High responders show differing O2 levels (peak O2 response) to 3 pellets compared to low responders in both the NAc and the OFC, but don’t really discuss possible reasons for this difference in the manuscript at all. I presume these sessions were conducted after PR training and data collection, therefore there is the possibility that group differences to the reward during these non-contingent reward delivery sessions are due to learning during the instrumental training sessions.  Therefore it might be interesting to see if these differences were the case before behavioral training.

b. I also think it might have been interesting if they had compared O2 responding in animals whose reward delivery was yoked to those on the PR task – as this could help compare/dissociate motor behaviour  and reward delivery from effort in another manipulation. I appreciate, however, that the authors already have a lot of data to deal with.


7. There are a number of analyses reported throughout the manuscript that the data for are not included in a figure/table, so it is hard to come to an informed conclusion about these results.  I’d at least like to see means/SEM for all analyses – especially as there are so many similar comparisons it can be hard to keep track. Eg. Delay response stratification – analyses reported on lines 410/411 where are the means for this analysis. OFC data did not predict PR performance – figures for these correlations are not shown (line 471 in discussion)

8. Discussion Line 515. The authors make an interesting point about prediction errors – using the same methodology they could also examine exepectation based on O2 response prior to the reward.

Additional questions
1. Rats received 5 days of progressive ratio (PR) training with the aim of stabilizing performance, and the 10 sessions in which the O2 response was recorded. As most of these analyses was conducted on the averages of these 10 sessions it would be interesting to report the variability in the PR data across these sessions – were some animals more consistent than others? What was the range of the breakpoint of these sessions for each animal?
2. Whole session amperometry analysis (Page 9 line 211 onwards) – this needs further clarification as it is not clear what exactly was done.
3. Why not compare fixed ratio responding at different work rates (high/low responders)? Unclear why the progressive ratio task is necessary to show these results.
4. P11 line 267 repetition of Sidak adjustments

____________________________________________________________________________________

Authors’ Response	14/08/18

_____________________________________________________________________________________

Dear Professors Bolam and Foxe

We would like to extend our gratitude for your consideration of our manuscript for publication in *The European Journal of Neuroscience* and for the time invested by yourselves, Dr Anthony Isles and the reviewers in preparing the constructive feedback, comments and suggestions we have received.

In response to these comments we have made a number of amendments to the content of the manuscript (underlined in the revised manuscript file) and detailed in our responses to the reviewers below. We have also formatted the figures and manuscript to be in-line with EJN guidelines.

Taken together, we believe these modifications have substantially strengthened the manuscript and enhanced readability. We hope that you and the reviewers find them satisfactory.

Many thanks again for your time,

Best wishes on behalf of all the authors,

Jonathan Hailwood

***Reviews:***

***Reviewer: #1***

***Comments to the Author***

***This is an interesting paper in which Hailwood and colleagues, showed that oxygen responses within the nucleus accumbens, but not the orbitofrontal cortex, were associated with individual differences in effort exertion in rats. To this aim the authors used constant potential in vivo amperometry in rats performing a progressive ratio task. Furthermore, the authors put in place adequate control to exclude possible confounds.***

***The experiments are well conceived and clearly described. Overall, the manuscript is well written and easy to follow. The authors provided an interesting and thorough discussion.***

***I have just a suggestion.***

***By shortening the discussion (thorough but lengthy), the authors should save some space where to discuss explicitly the study into the RDoC perspective (dedicated section). I believe that this already excellent study would benefit from it.***

Thank you for this suggestion, we have added a discussion of the significance of the study within the RDoC framework (p25).

***Reviewer #2
Comments to the Author***

***The authors report an interesting study examining oxygen responses in the nucleus accumbens(NAc)  and orbital frontal cortex (OFC) during progressive ratio (PR) task in rats, demonstrating increased oxygen response in the NAc which they conclude is a consequence of increased effort on the task. I think the topic is of interest to the audience of EJN but the results section in particular can get a bit confusing. The authors report several analyses with many different behavioural and O2 measures, and it is not always clear what data the analyses are conducted on. This, combined with errors and omissions in the data makes the findings hard to assess appropriately.  I believe, however, that with further revision the manuscript would be suitable for publication***

***1.	Cannulae were implanted into 2 regions of the OFC unilaterally. Why were these not bilateral implantations. Moreover the different cannulae implantations  into medialorbital and lateral regions were not used – instead recordings from both were combined (although there are fewer in the lateral OFC). Why was this the case? And are there any differences in recording from the regions of the OFC? This needs to be addressed in the manuscript.***

A potential criticism of studies investigating event-related, regional O_2_ activity is that the changes seen were part of some global confound, that may be due to movement or arousal. This may be especially problematic when using PR schedules given the highly energetic responding that rats typically engage in.

Therefore, it was important to demonstrate a degree of regional specificity to the results. We chose the orbitofrontal cortex primarily as a control brain region. Whereas there are some reports of differences in the function of the medial and lateral orbitofrontal cortices, in the present study the numerical pattern of the O_2_ reward responses was very similar for these two areas. There also appeared to be little difference in behavioural performance between rats with electrodes implanted in either region. Unilateral electrode implantation has been used in previous oxygen amperometry studies (e.g. Li *et al*., 2015). We therefore, felt that was appropriate to combine these regions to increase the power of the study, particularly as the OFC is typically treated as a single region in human fMRI studies

We have added a discussion of these factors to the manuscript (p24)

2.	***Stability of behavioural performance ( p13 and Figure 2)***

***The authors report that high responders perform more lever presses during both early and late trials than low responders, but there were no reported differences between high/low responders on delay to reward, or on lever press/sec  – I’m not sure how this can be the case if the high responders are responding more? The high responders are at a higher breakpoint (FR) in which case there should be a longer delay to reward if responding at the same rate as the low responders. Unless (p10 line 233) median splits are performed separately for each measure – although I’m not sure of the logic for this. It is also important to also report the breakpoints of these groups rather than just the mean lever presses.***

It would be intuitive to think that low responders would have shorter delays to rewards due to a lower mean response requirement. However, the difference in breakpoint between groups, although statistically significant, was relatively small. This was a consequence of the overall low number of trials completed by the rats in this study. We have added a figure to illustrate this finding (figure 2B). Furthermore, we found that, due to pauses in responding within some ratios (particularly late ratios), there was a high degree of variance in the delay to reward. Some have suggested that the length of this pre-reinforcement pausing is associated with an animal’s motivational state (e.g. Brackney, R. J., et al., (2011). *J Exp Anal Beh*, *96*(1), 17-38), therefore it is not surprising if low responding rats show a tendency to show longer pauses in responding. Together this pausing, coupled with the low breakpoints, masked any group differences in delay-to-reward caused by the different in the response requirements.

Median splits were performed upon the delay- and response rates to compare, oxygen but not behavioural differences between groups. We have clarified the text, to better explain this (p13).

***3.	Within session O2 signal stability –p14 line 319 onwards.***

***The authors report NAc O2 recordings from a total of 143 PR sessions (from 15 rats), wheres OFC reacordings were from 83 sessions (and only 10 rats). Why were there differences in sessions/rats from each of the regions? And did all 10 animals contribute to recordings from both regions of the OFC?***

***In addition – the behavioural data reported was for all 10 sessions, for all 15 rats – was there any variability in behavioural performance when you use only the sessions that were included in the O2 analysis?***

We had to exclude OFC recordings from a number of rats due to misplaced electrodes, this was the reason for the differences in the number NAc and OFC rats. Furthermore, the data from some electrodes in some sessions had to be excluded due to artefacts in the data signal. In these cases, the behavioural data from these individual rats was also excluded. Therefore, these sessions were not used in calculating, for example, the high and low response groups. We have modified the text to clarify this point (p14). The majority of rats only had either the medial or the lateral electrode correctly placed. We have added these numbers to the text (p13)

***4.	Early vs Late Trial stratification p14 line 330 onwards.***

***Has the sub-baseline O2 response been observed in other studies? Or is the baseline not meaningful in early trials due to greater variability in behavioural response?***

The present sub-baseline response is likely an artefact of the task design. As the baseline is calculated from a 1s pre-reward period the O2 levels were likely already elevated as a consequence of the lever pressing and the return to normal levels appears as a sub-baseline, negative response. The lack of a sub-baseline response in the late trials may be due to a greater magnitude O2 response to reward when the effort expended to obtain the reward was higher.

The majority of O2 amperometry studies do not use such designs, and therefore do not report such negative responses. However, sub-baseline O2 responses have been observed in previous studies (e.g. McHugh et al., 2014; Francois et al., 2014).

***5.	Low vs High response stratification p15. I think you are missing some figures, or there is an error – Para starting p17 line 393 references figure 4G and 4H as showing the data for the OFC, however these figures have already been referred to in line 391 as showing data from the NAc for the first 5 trials of the session, thus these data are hard to interpret.***

Thank you for noticing this duplication error, we have corrected the text.

***6.	The effects of non-contingent reward delivery on O2 levels was examined – specifically comparing the effect of 1 vs 3 pellet delivery – to account for non-specific effects of arousal .***

***a.	The authors report that High responders show differing O2 levels (peak O2 response) to 3 pellets compared to low responders in both the NAc and the OFC, but don’t really discuss possible reasons for this difference in the manuscript at all. I presume these sessions were conducted after PR training and data collection, therefore there is the possibility that group differences to the reward during these non-contingent reward delivery sessions are due to learning during the instrumental training sessions. Therefore it might be interesting to see if these differences were the case before behavioral training.***

Thank you for this point, we have proposed a potential explanation for this in the following discussion (p23)

“When examining the effects of noncontingent reward delivery in NAc O2 responses, it appears that only the high responding rats show an increased magnitude O2 response following the three-pellet reward delivery (figure 5B). It is worth noting that high responders appeared to show a greater magnitude reward response only following three pellet rewards. Since this probe was conducted after PR training, animals had only been exposed to single pellet rewards. The enhanced increase to these unexpectedly large rewards, may be a larger positive prediction error, which may reflect individual differences within the mesolimbic DA system. Furthermore, during this probe, rewards were delivered independently of any instrumental contingency, suggesting the O2 responses reflect Pavlovian influences upon reward. The present association between NAc O2 responses and individual differences in PR performance may, therefore, reflect individual differences in Pavlovian influences on behaviour or incentive motivation. In other words, the greater incentive motivation enabled the high performing rats to overcome greater effort costs to obtain more rewards under the PR schedule of reinforcement”.

***b.	I also think it might have been interesting if they had compared O2 responding in animals whose reward delivery was yoked to those on the PR task – as this could help compare/dissociate motor behaviour  and reward delivery from effort in another manipulation. I appreciate, however, that the authors already have a lot of data to deal with.***

This would have been an interesting manipulation. Although we seek to address the potential confounds of escalating delays, the use of a yoked-reward group would be the best way to examine the effects of delays upon NAc O2 reward responses, in the absence of changes in effort-requirements. This is something a future study should examine. We have amended to discussion to mention this (p22)

***7.	There are a number of analyses reported throughout the manuscript that the data for are not included in a figure/table, so it is hard to come to an informed conclusion about these results.  I’d at least like to see means/SEM for all analyses – especially as there are so many similar comparisons it can be hard to keep track. Eg. Delay response stratification – analyses reported on lines 410/411 where are the means for this analysis. OFC data did not predict PR performance – figures for these correlations are not shown (line 471 in discussion)***

Thank you for this point, we have added tables containing the data that are not included in the figures to ease interpretation of the results

***8.	Discussion Line 515. The authors make an interesting point about prediction errors – using the same methodology they could also examine exepectation based on O2 response prior to the reward.***

This is an interesting point. Reward expectations have been investigated in prior studies using O2 amperometry. We have added a brief mention of these to the discussion (p23)

***Additional questions***

***1.	Rats received 5 days of progressive ratio (PR) training with the aim of stabilizing performance, and the 10 sessions in which the O2 response was recorded. As most of these analyses was conducted on the averages of these 10 sessions it would be interesting to report the variability in the PR data across these sessions – were some animals more consistent than others? What was the range of the breakpoint of these sessions for each animal?***

The behavioural variability in these sessions had been reported in the Behavioural Analysis section of the results. There were no significant differences in the group-level breakpoint between any session. Additionally, the within-subject variability was small. We have added a figure showing the variability in breakpoint to ease interpretation (Figure 2)

***2.	Whole session amperometry analysis (Page 9 line 211 onwards) – this needs further clarification as it is not clear what exactly was done.***

We have amended the description to better explain the whole session analysis (p9):

“As slow drifts in baseline over the course of a session may confound event-related analyses, global changes in O_2_ levels following session start were assessed. We therefore examined the temporal profile of O_2_ signals across the course of a PR session, to assess for any absolute changes in global current in either the NAc or OFC. Following the first lever presentation, the change in current was divided into 120s non-overlapping bins. Animals are typically only actively engaging in the task for a portion of the 45-minute PR session (i.e. the period prior to breakpoint being reached). Therefore, the absolute change in global O_2_ levels was examined only for the rats’ mean active period, which was calculated by as the time from session onset to the completion of the final trial within a session”

***3.	Why not compare fixed ratio responding at different work rates (high/low responders)? Unclear why the progressive ratio task is necessary to show these results.***

The use of fixed ratio schedules could be used to assay effort and we would expect to produce the same associations between NAc O2 reward responses and effort exerted. It could also be argued that FR schedules, if used appropriately could better control for the confounds of escalating delays observed in PR tasks. However, we chose this PR task in the present study as such schedules are used across species (including healthy humans and clinical populations) as assays of motivation. As a purpose of this study was to identify a translatable imaging correlate of motivation, it was reasonable to also use a translatable assay of behaviour.

***4.	P11 line 267 repetition of Sidak adjustments***

*Thank you for this observation, we have removed this repetition.*
